# Network component analysis provides quantitative insights on an Arabidopsis transcription factor-gene regulatory network

**DOI:** 10.1186/1752-0509-7-126

**Published:** 2013-11-14

**Authors:** Ashish Misra, Ganesh Sriram

**Affiliations:** 1 Department of Chemical and Biomolecular Engineering, University of Maryland, College Park, MD 20742, USA; 2 Current affiliation: DBT-ICT Centre for Energy Biosciences, Institute of Chemical Technology, Mumbai, India

## Abstract

**Background:**

Gene regulatory networks (GRNs) are models of molecule-gene interactions instrumental in the coordination of gene expression. Transcription factor (TF)-GRNs are an important subset of GRNs that characterize gene expression as the effect of TFs acting on their target genes. Although such networks can qualitatively summarize TF-gene interactions, it is highly desirable to quantitatively determine the strengths of the interactions in a TF-GRN as well as the magnitudes of TF activities. To our knowledge, such analysis is rare in plant biology. A computational methodology developed for this purpose is network component analysis (NCA), which has been used for studying large-scale microbial TF-GRNs to obtain nontrivial, mechanistic insights. In this work, we employed NCA to quantitatively analyze a plant TF-GRN important in floral development using available regulatory information from AGRIS, by processing previously reported gene expression data from four shoot apical meristem cell types.

**Results:**

The NCA model satisfactorily accounted for gene expression measurements in a TF-GRN of seven TFs (LFY, AG, SEPALLATA3 [SEP3], AP2, AGL15, HY5 and AP3/PI) and 55 genes. NCA found strong interactions between certain TF-gene pairs including LFY → *MYB17*, AG → *CRC*, AP2 → *RD20*, AGL15 → *RAV2* and HY5 → *HLH1*, and the direction of the interaction (activation or repression) for some AGL15 targets for which this information was not previously available. The activity trends of four TFs - LFY, AG, HY5 and AP3/PI as deduced by NCA correlated well with the changes in expression levels of the genes encoding these TFs across all four cell types; such a correlation was not observed for SEP3, AP2 and AGL15.

**Conclusions:**

For the first time, we have reported the use of NCA to quantitatively analyze a plant TF-GRN important in floral development for obtaining nontrivial information about connectivity strengths between TFs and their target genes as well as TF activity. However, since NCA relies on documented connectivity information about the underlying TF-GRN, it is currently limited in its application to larger plant networks because of the lack of documented connectivities. In the future, the identification of interactions between plant TFs and their target genes on a genome scale would allow the use of NCA to provide quantitative regulatory information about plant TF-GRNs, leading to improved insights on cellular regulatory programs.

## Background

Gene expression is a complex process regulated by the interactions of proteins and other molecules with genes. This regulation occurs at multiple levels, giving rise to gene regulatory networks (GRNs) that define the regulatory programs for the expression of specific genes in response to specific cues [1]. One of the biggest challenges of systems biology is deciphering the organization of GRNs [2,3]. This task is further complicated by feedback- and feedforward-type interactions of a multitude of genes and their protein products upon themselves and others. GRNs are usually modeled as graphs with nodes representing system components (e.g. molecules) and edges indicating interactions between components [1,4,5]. Various methodologies have been developed for the analysis of GRNs including directed graphs, Boolean networks, Bayesian networks and differential equations [2,6-11]. An important subset of GRNs models gene expression as a result of the action of transcription factors (TFs) upon their target genes. In these models, directed edges from TFs to their target genes represent transcriptional regulation, and constitute a hierarchical network governing gene expression [2,12]. The reconstruction of TF-GRNs involves the identification of genes that encode the TFs and the identification of the target genes of the TFs.

There is a considerable amount of information available on TF-gene interactions in microbes which is housed in databases. For example, RegulonDB and DBTBS are extensively curated databases containing information on transcriptional regulation in the bacteria *Escherichia coli* and *Bacillus subtilis* respectively [13,14]. The RegPrecise database contains similar information for many other prokaryotes [15], as does the YEASTRACT database for *Saccharomyces cerevisiae*[16]*.* The availability of such resources permits accurate reconstruction of TF-GRNs, and consequent network analyses to obtain insights on regulatory capabilities of the organism of interest. For plants, such information is comparatively sparse, with most regulatory studies directed at inferring GRNs in isolated organs such as roots or leaves, or processes such as development or abiotic stress response [9,17,18]. Large-scale TF-gene interaction data are only available for *Arabidopsis thaliana* and housed in the Arabidopsis Gene Regulatory Information Server (AGRIS) [19].

Although the establishment of TF-GRN connectivity (i.e. which TF regulates which gene) is very useful, the information contained in such connectivity maps is binary and not quantitative. Understanding quantitative changes in gene expression would provide deeper insights into gene regulation and perhaps even enable predictive modeling of cellular regulatory programs. This would, however, require significant mathematical processing of high-throughput gene expression datasets [20]. Under a given condition, gene expression would depend on the strength of the interaction between a TF and its target gene as well as the activity of the TF at that condition. Therefore, given the connectivity of a TF-GRN and gene expression values under a set of conditions, the next set of questions that need to be answered are: (i) Is it possible to obtain connectivity strengths (CS) of TF-gene interactions for the network and (ii) Can we quantify how TF activity varies across conditions? Estimating the CS between a TF and its target gene may be possible computationally by determining the decrease in free energy for binding between the TF and the DNA region of the target gene it binds to [21,22]. A higher free energy change would indicate stronger binding and a lower free energy change weaker binding [21,23]. However, thermodynamic calculations for determining changes in free energy are nontrivial and would require knowledge of binding thermodynamics of many TFs and their target genes. The CS between a TF and a gene can also be determined experimentally by using binding assays for determining parameters such as the dissociation constant or changes in free energy and enthalpy [24]. Although parameters derived from such TF-gene binding assays are available in some databases, it would be a laborious exercise to obtain these values for every TF-gene pair [25]. For estimating changes in TF activity, experimental assays may be employed based on the binding of the active form of the TF with a target reporter molecule. However, such assays are only available for a limited number of TFs and would have to be conducted for each condition. Additionally, the experimental approaches for determining TF-gene CS and TF activities suffer from the drawback of being *in vitro* studies. Consequently, the values determined may not represent the *in vivo* interactions of the TFs and genes wherein multiple TFs can act on a single gene. It may appear that changes in the expression levels of the genes corresponding to the TFs could be used as surrogates for TF activities. However, a shortcoming of this approach is that TF activity could be considerably affected by post-transcriptional and post-translational modifications such as phosphorylation and acetylation, and can therefore, differ substantially from the expression levels of corresponding genes.

To deduce such quantitative information about TF-GRNs, researchers have developed methodologies like network component analysis (NCA) and regulatory element detection using correlation with expression (REDUCE) [26-29]. NCA, in particular, models gene expression to be the result of the connectivity strength between TF-gene pairs and TF activity [26]. The strength of the TF-gene interaction indicates the extent of the control of a TF over the transcription of a target gene, whereas the TF activity quantifies how active the TF is in regulating its target genes either via activation or repression. NCA uses connectivity information about the underlying network and gene expression data to obtain non trivial information about TF activity and TF-gene connectivity strength. Because the TF activity provides a measure for the TF in its final state, it includes information about the post-transcriptional and post-translational modifications. Compared to experimental approaches for obtaining similar information, NCA allows the deduction of such important regulatory information by a much simpler approach involving the measurement of gene expression for the set of genes in a network. The other input for NCA, the connectivity between TFs and genes, is available for many organisms in databases. Consequently, NCA provides an additional layer of regulatory information without the use of sophisticated experimental measurements [28].

Given the connectivity map underlying a TF-GRN, the NCA framework allows the decomposition of gene expression data into TF activities and connectivity strengths (CS) between each TF and its target genes. NCA models TF regulation of gene expression by the matrix equation [26,27]:

(1) $${\left[\log\;G\right]}_{m\times n}={\left[\mathrm{CS}\right]}_{m\times p}\times {\left[\log\;\mathrm{TFA}\right]}_{p\times n}$$

Here, [**G**]_*m*×*n*_ is a matrix representing an experimental gene expression dataset consisting of the expression of *m* genes across *n* conditions; [**log G**]_*m*×*n*_ is its log-transformed version. Similarly, [**TFA**]_*p*×*n*_ is a matrix of the activities of *p* TFs across the *n* conditions; [**log TFA**]_*p*×*n*_ is its log-transformed version. These two matrices are linked by [**CS**]_*m*×*p*_, which consists of the CS of *p* TFs on *m* genes.

The log-linear relationship used in NCA allows the benefits of linearization during the decomposition while capturing non-linear network behavior to a limited extent. Besides, since high-throughput gene expression data are usually expressed relative to a control condition, the log-linear relationship is convenient while working with relative gene expression data [26]. The NCA decomposition is unique up to a scaling factor, when the [**CS**] and [**TFA**] matrices satisfy a set of criteria termed “NCA-compliance” criteria [26]. The originally reported NCA algorithm [26] required the presence of as many gene expression data points as regulators for the decomposition. However, a more recent modification of that algorithm [30] permits the analysis of limited microarray datasets, thus widening the applicability of NCA. A detailed analysis of the original NCA algorithm and the modified algorithm are provided in the respective publications [26,30].

NCA has been previously applied for the analysis of microbial and mammalian transcriptional networks. Liao et al. [26] first used NCA to study cell cycle regulation in *S. cerevisiae*, and specifically to quantify the activities of different TFs during various stages of the cell cycle, thus gaining insight on the regulatory roles of specific TFs at each stage. Kao et al. [27] investigated the effect of a glucose-to-acetate carbon source transition on the activity of TFs in *E. coli.* They observed specific trends in the changes in activities of several TFs (CRP, FadR, IclR, and Cra) important during this transition. In a further extension of this study, they investigated the growth lag that resulted by the deletion of the *ppsA* gene in *E. coli* during this carbon source transition [28]. By using NCA, they deduced the activities of TFs that were affected by the deletion and proposed a mechanism for explaining the growth lag. A set of twin studies investigating the effect of the reactive nitrogen species, nitric oxide and S-nitrosoglutathione, on *E. coli* identified important TFs involved in response to the respective treatments [31,32]. The first study identified 13 important TFs of which ten have not been previously documented to be involved in response to nitric oxide [31]. The subsequent study with S-nitrosoglutathione identified four novel TFs (CysB, SF, FlhDC, and TTA) involved in response to the treatment [32]. The use of NCA in combination with transcriptome data allowed the construction of models depicting the response process for both studies. Brynildsen et al. investigated the isobutanol response network in *E. coli* and identified the ArcA-ArcB system to be a major regulator of the response via a loss of quinone function [33]. They also compared differences in TF activities in response to isobutanol with those seen for butanol and ethanol, and identified 6 TFs with differing activities for butanol, and 19 TFs with differing activities for ethanol compared to isobutanol. In another study [34], Buescher et al. performed genome wide TF-gene analysis of B. subtilis during a change in carbon substrate from glucose to malate and vice versa, and determined CS for 2900 TF-gene interactions. They deduced TF activities for 154 TFs out of which 127 TFs were found to change their activities significantly. Interestingly, many of these changes in TF activity were not seen at the mRNA level thus implicating the role of posttranslational modifications for the changes in TF activities. In mammalian systems, Sriram et al. studied the effect of overexpressing the glycerol kinase gene in rat hepatoma cells using a network of 62 genes and 9 TFs [35]. They found an increase in the TF activity for 7 of the TFs (ChREBP, Sp1, HNF1α, HNF4α, PPARα, LXRα, and glucocorticoid receptor [GR]) and a decrease in activity for the remaining 2 TFs (SREBP1a and CEBPβ). The increased activity of GR was hypothesized to be a result of the moonlighting nature of the glycerol kinase enzyme [36]. Sriram et al. experimentally verified the NCA-deduced change in TF activity of GR in the glycerol kinase-overexpressing cell line, thus demonstrating the power of NCA for deducing TF activities from gene expression data in a mammalian network. In a recent study [37], Tran et al. studied the TFs directly downstream of PTEN (phosphatase and tensin homologue deleted on chromosome 10), which is an important tumor suppressor gene. They identified 20 TFs whose activities were altered significantly by the expression of PTEN even when the mRNA levels of the corresponding genes did not alter significantly. They found many of the identified TFs varied in murine and human cancer models, and provided a signature for identifying the status of PTEN in cancers caused by PTEN loss.

In this article, we report the application of NCA on a plant TF-GRN using available regulatory information from AGRIS. Starting with a set of TFs known to be important in floral development, we mined AGRIS to establish a network consisting of confirmed TF-gene connectivities in this developmental event. We used previously published gene expression data [38] for four types of cells isolated from the shoot apical meristem, which is known to initiate the growth of floral organs. By using the connectivity information and gene expression datasets, we used NCA to deduce activities for the NCA-compliant TFs, and numerical values of CS between the TFs and their target genes. To the best of our knowledge, this is the first study to apply NCA to dissect a plant TF-GRN.

## Results

**Figure 1:**
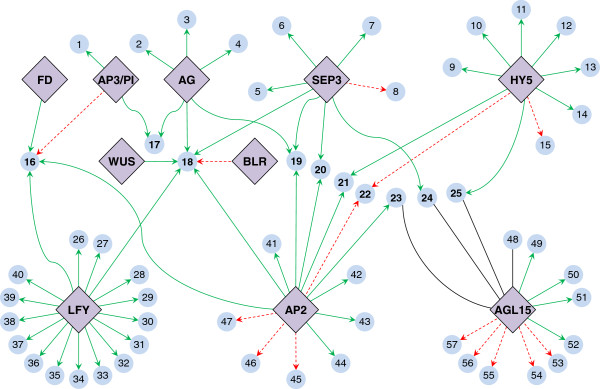
**Initial connectivity map of TF-gene interactions documented in AGRIS.** Connections between 10 TFs (violet) important in floral development and their 57 target genes (light blue). Edges from TFs to genes indicate target genes of TFs: a solid green edge indicates gene activation, a dashed red edge indicates gene repression and solid gray edges indicate an unknown interaction. *[The gene abbreviations and the corresponding Arabidopsis gene model are: 1 - NAP (At1g69490), 2 - CRC (At1g69180), 3 - GIK (At2g35270), 4 - APL (At2g27330), 5 - AGL2 (At5g15800), 6 - AGL4 (At3g02310), 7 - AGL8 (At5g60910), 8 - AGL3 (At2g03710), 9 - ACS8 (At4g37770), 10 - ADR1 (At1g33560), 11 - INV1 (At1g02810), 12 - UNK4 (At1g56660), 13 - FAD bin (At1g57770), 14 - UNK5 (At2g25460), 15 - UNK6 (At2g25890), 16 - AP1 (At1g69120), 17 - FLO10 (At3g23130), 18 - AG (At4g18960), 19 - AGL5 (At2g42830), 20 - AGL1 (At3g58780), 21 - HLH1 (At2g42870), 22 - RD20 (At2g33380), 23 - EDF4 (At1g13260), 24 - AGL22 (At2g22540), 25 - RAV2 (At1g68840), 26 - ACR7 (At4g22780), 27 - ASN1 (At3g47340), 28 - BGLU15 (At2g44450), 29 - BZIP (At1g68880), 30 - AGL10 (At1g26310), 31 - UNK1 (At5g03230), 32 - LEA (At3g52470), 33 - UNK2 (At1g61830), 34 - LEU1 (At5g49770), 35 - HB51 (At5g03790), 36 - GRA1 (At3g19390), 37 - UNK3 (At5g60630), 38 - MYB17 (At3g61250), 39 - SUS4 (At3g43190), 40 - TLP8 (At1g16070), 41 - APUM9 (At1g35730), 42 - DAN1 (At3g04620), 43 - KIN1 (At1g11050), 44 - AGL44 (At2g14210), 45 - PERK4 (At2g18470), 46 - DNA1 (At3g47680), 47 - PKS2 (At1g14280), 48 - AGL25 (At5g10140), 49 - FUS3 (At3g26790), 50 - IAA30 (At3g62100), 51 - LEC2 (At1g28300), 52 - ATGA2OX4 (At1g02400), 53 - LEA7 (At1g52690), 54 - CSP4 (At2g21060), 55 - AGL18 (At3g57390), 56 - DTA2 (At2g45830), 57 - CBF2 (At4g25470)].*

In this work, we tested the ability of NCA to quantitatively deduce nontrivial information about a plant TF-GRN solely from gene expression data and previously documented TF-gene connectivities. Toward this, we established a TF-GRN consisting of ten TFs: LEAFY (LFY), AGAMOUS (AG), SEPALLATA3 (SEP3), APETALA2 (AP2), AGAMOUS-LIKE 15 (AGL15), ELONGATED HYPOCOTYL 5 (HY5), APETALA3/PISTILLATA (AP3/PI), ATBZIP14 (FD), WUSCHEL (WUS) and BEL1-LIKE HOMEODOMAIN 9 (BLR) using regulatory information available in AGRIS. The network included 57 genes known to be regulated by these TFs, as listed in the AtRegNet database from AGRIS [19]. On the basis of the interaction information obtained from AGRIS (Additional file 1, sheet: AGRIS TF-gene verification), we constructed an initial connectivity matrix for this network for use in NCA (Additional file 1, sheet: Initial connectivity matrix). We screened the Botany Array Resource [39] to locate pertinent gene expression data for the TFs under consideration. From this database, we selected microarray data from a study [38] that sampled four distinct types of shoot apical meristemmatic cells (denoted as CLV3n, CLV3p, FILp and WUSp) and that showed expression of the genes encoding LFY and other TFs included in our network (Additional file 1, sheet: Original microarray data). We then employed the NCA toolbox [26,30] to analyze the network using the gene expression data and the initial connectivity matrix, assuming that the CS was the same across all four cell types. Initial networks constructed for NCA have to be pruned to make them NCA-compliant [26,30]. On these lines, a subnetwork of 55 genes and 7 TFs (Figure 1) was found to be NCA-compliant (Additional file 2, sheet: NCA-compliant network). The entire NCA output along with comparisons between deduced TF activities and the expression levels of the genes encoding the TFs, is included in Additional file 2.

### NCA deduces the strengths of TF-gene interactions

**Figure 2:**
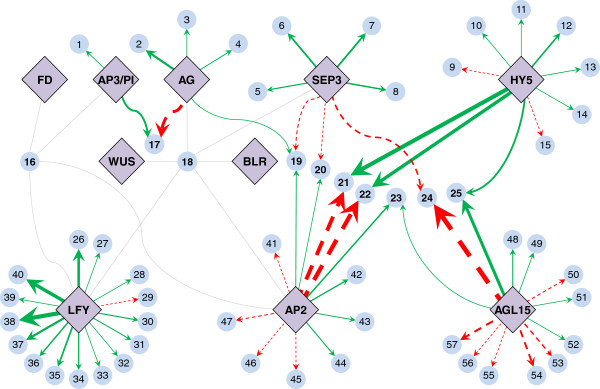
**Connectivity map of TF-gene interactions as deduced by NCA.** Connections between 10 TFs (violet) important in floral development and their 57 target genes (light blue), as deduced by NCA. Edges from TFs to genes indicate target genes of TFs: a solid green edge with an arrow indicates gene activation by a TF, a dashed red edge with an arrow indicates gene repression by a TF and solid gray edges indicate an undeterminable interaction. Edge thickness is proportional to the TF-gene CS deduced by NCA. *[The gene abbreviations and gene model for the genes are the same as those used in Figure*1*].*

NCA decomposes the gene expression matrix into two components: a matrix of [**CS**] signifying interactions between TFs and their target genes, and a matrix [**log TFA**] of TF activities (Eq. {1}). The matrix decomposition applies specific scaling factors for the activity of a given TF as well as the CS between that TF with its target genes. If negative, this scaling factor can invert the sign of the TF activity and CS pertaining to a given TF. Consequently, the CS and TF activity for each TF may need to be corrected by comparing the CS with the initial connectivity matrix and specifically looking at the connectivity between a TF and gene that is convincingly known from experimental evidence. Based on this comparison, we corrected the CS and corresponding TF activity for AG, SEP3, AP2 and HY5 (Additional file 2, sheet: TFA and mRNA). Figure 2 depicts the deduced CS values in the analyzed network. The CS between a TF and its target gene determines how strongly the TF activates or represses the corresponding target gene. We used two criteria for defining strong interactions (i) A CS of more than +1 (activation) or less than −1 (repression) (ii) Low variability across multiple NCA replicate runs. The CS used for distinguishing strong from non-strong interactions is arbitrary but allows a means for distinguishing interactions between TFs and genes. For example, LFY is strongly connected to *ACR7, HB51, GRA1, UNK3, MYB17, TLP8* and weakly connected to *ASN1, BGLU15, BZIP, LEA, UNK2,* and *SUS4* among its target genes. Other sets of strong interactions include the following pairs: AG → *CRC*; SEP3 → *AGL4*; SEP3 → *AGL3*; SEP3 → *AGL8*; AP2 → *HLH1*; AP2 → *RD20*; AGL15 → *AGL22*; AGL15 → *LEA7*; AGL15 → *RAV2*, AGL15 → *CSP4*; AGL15 → *CBF2*; HY5 → *HLH1*; HY5 → *RAV2*; HY5 → *RD20*; HY5 → *UNK4* and AP3/PI → *FLO10*.

### Gene expression levels simulated by NCA agree well with the originally measured gene expression levels

**Figure 3:**
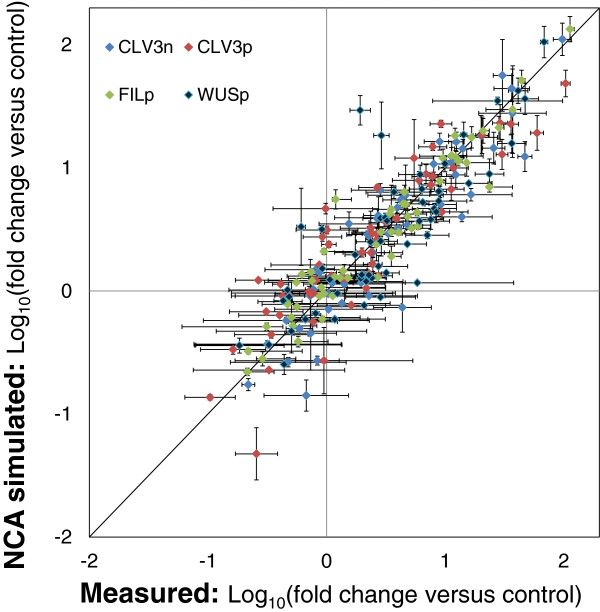
**Comparison between gene expression (mRNA) levels calculated by NCA and (previously) measured by microarray analysis.** From measured gene expression values (matrix [log_10_G]) across four cell types (conditions), NCA deduced the unknown TF activities (matrix [log_10_TFA]) and the TF-gene CS (matrix [CS]) in Eq. {1} for seven NCA-compliant TFs. Based on this, the gene expression values simulated by NCA were calculated as the product of the TF-gene CS (matrix [CS]), and the TF activities (matrix [log_10_TFA]). This plot compares the NCA simulated gene expression values with experimental gene expression levels for the four cell types. A good match was seen between both (R^2^ = 0.816) depicting the ability of NCA model to account for the gene expression measurements. The horizontal error bars are from replicates for the measured gene expression across all 4 cell types, the vertical error bars are from the corresponding replicates of NCA simulated gene expression values.

We obtained the gene expression values simulated by NCA by multiplying the [**CS**] matrix with the [**log**_**10**_**TFA**] matrix for each of the four cell types (Eq. {1}). A comparison of the NCA-simulated gene expression levels with the original measurements as obtained by Yadav et al. [38] by microarray analysis, shows a good agreement between the two sets (Figure 3). Some discrepancies were seen in the NCA-simulated gene expression levels, which may be attributable to residues arising in the least-squares minimization during the NCA decomposition.

### TF activities deduced for LFY, AG, HY5 and AP3/PI agree well with expression levels of genes encoding these TFs

**Figure 4:**
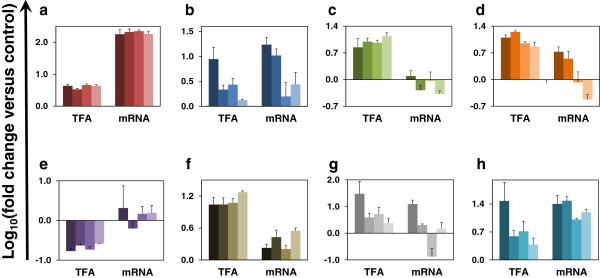
**Comparison between TF activities and expression levels of genes encoding the TFs.** TF activities were deduced by NCA for seven NCA-compliant TFs and compared with the expression levels of genes encoding the TFs across all four cell lines. For each TF panel **(a)** LFY **(b)** AG **(c)** AP2 **(d)** SEP3 **(e)** AGL15 **(f)** HY5 **(g)** AP3 **(h)** PI, values are indicated in a different color with the cell lines CLV3n, CLV3p, FILp and WUSp shown from left to right with decreasing shades. Good agreement between the direction of TF activity and mRNA change (relative to control) is apparent for most TFs except AP2, AGL15 and SEP3.

NCA provides log-fold changes of the TF activities with respect to a control condition. We compared changes in the TF activity across the four cell types with respect to a control by plotting the activities for the seven TFs against the corresponding gene expression values (Figure 4). For instance, the consistent gene expression level of *LFY* across all four cell types agreed with the deduced TF activity for LFY, which was also consistent across the four cell types (Figure 4a). AG exhibited a decreasing trend of TF activity across the four cell types with CLV3n showing the highest activity. This trend also appeared in its gene expression values (Figure 4b). For HY5, the TF activity remained nearly unchanged across all four cell types while the gene expression showed smaller changes for CLV3n and FILp compared to CLV3p and WUSp (Figure 4f). The AP3/PI TF had higher activity in the CLV3n cells and a lower change in activity in the other three cell types. Because AP3 and PI proteins co-regulate the activity of some genes, we compared the activity of the AP3/PI TF separately with the *AP3* and *PI* genes (Figure 4g & 4h). Interestingly, the TF activity trend of AP3/PI agreed better with the gene expression of *PI*, whereas *AP3* expression showed an opposite trend for the FILp cell type. The TF activity of SEP3 showed agreement with its gene expression levels for two cell types (CLV3n and CLV3p), and a discrepancy for the other two cell types (FILp and WUSp) (Figure 4d). Two TFs, AP2 and AGL15, had differing trends in their TF activities and gene expression levels (Figure 4c & 4e). This may be explained by the large biological errors of the gene expression levels of both *AP2* and *AGL15*, which were comparable to the measurements. Further, we analyzed the changes in TF activities across the cell types statistically by comparing individual pairs of cells using a p-value cutoff of 0.05. The TF activities deduced by NCA for AG and SEP3 showed variation across multiple cell type pairs, while SEP3 and AP3 showed similar variation in their mRNA levels.

### Normalized plots of TF activities and gene expression values showed a good fit for LFY, AG, HY5 and AP3

**Figure 5:**
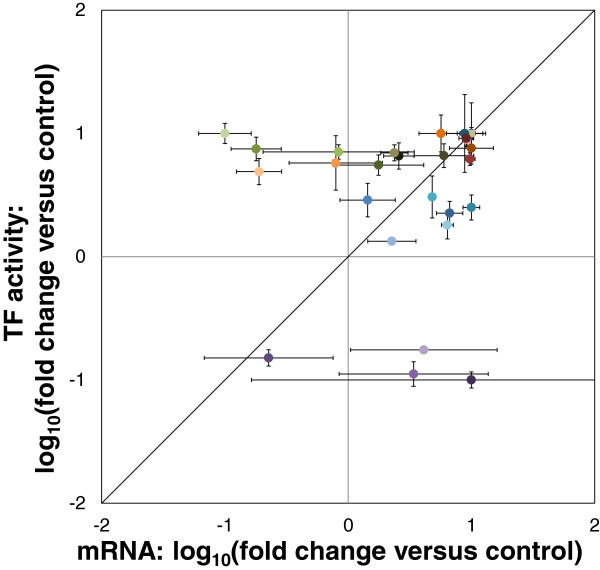
**Parity plot of normalized TF activities and expression levels of genes encoding the TFs.** TF activities were deduced by NCA for seven NCA-compliant TFs and compared with the expression levels of genes encoding the TFs across all four cell lines (Colors used for TFs correspond to those used in Figure 4). Good correlation is apparent for most TFs, but poor correlation is evident especially for AP2 and AGL15. The general agreement between normalized TF activity and expression level of the corresponding gene indicates the strength of NCA for deducing TF activities.

Our comparison of NCA-simulated TF activities and expression levels of the genes encoding the TFs allowed a qualitative comparison between the trends shown by the computational NCA and the experimental transcriptome analysis. To provide a better comparison between the TF activity and gene expression values for corresponding TFs, we normalized the values across all four cell types and prepared a parity plot by using maximum and minimum values across each set as the basis for normalization (Figure 5). This plot shows that TF activities deduced by NCA agreed well with expression levels of the TF-encoding genes, with only AP2 and AGL15 being exceptions.

## Discussion

TF-GRNs, which model interactions between TFs and their target genes, are an important class of cellular networks that define regulatory programs leading to gene expression [2,12]. TF-GRNs provide Boolean information about the regulation of genes by TFs, with meticulously compiled data available in databases like RegulonDB, YEASTRACT and AGRIS [13,16,19]. To deduce further quantitative information about the connectivities between TFs and their target genes, methodologies such as NCA and REDUCE have been developed [26,29]. Given the underlying network connectivity information, NCA can provide information on the connectivity strength between a TF and its target gene as well as the TF activity by using gene expression data [26,30,40]. Through such nontrivial, quantitative information, NCA can provide important parameters about a TF-GRN. In this study, we sought to apply the NCA approach to analyze a network comprising TFs important for floral development and their targets using underlying connectivity information available in the AGRIS database.

Floral development is one of the best characterized processes in plants with multiple studies providing much information at the molecular genetic level [41-43]. The most widely used model for explaining the initial development of the organs of a flower is the ABC model and its variants [42]. The model predicts floral development to result from the concerted action of multiple TF-encoding genes. For this study, we constructed a plant TF-GRN consisting of ten TFs, known to be involved in floral development, (LFY, AG, SEPALLATA3 (SEP3), AP2, AGL15, HY5, AP3/PI, FD, WUS and BLR) and 57 target genes with verified interactions obtained from AGRIS. LFY is known to be a master TF that regulates important events in the transition from vegetative to reproductive growth, and has another important role in the activation of floral homeotic genes [44-46]. Some of its downstream targets are known to be TFs that are important in flower morphogenesis. The other TFs included in our original network are important factors in floral development: AG, SEP3 and AGL15 are MADS domain TFs; AP2 belongs to the AP2/EREBP (ethylene responsive element binding protein) class of TFs; HY5 and FD are basic leucine zipper TFs that regulate flower development; AP3/PI is a member of the NAC TF family that is expressed in floral primordia and WUS and BLR are homeobox TFs [47]. We were unable to include some of the other TFs (AP1, FT and AGL20) important in the process due to a lack of sufficient confirmed targets for them in AGRIS for NCA compliance. We used gene expression data from a study by Yadav et al. [38] that analyzed the expression patterns across four different types of cells (named CLV3n, CLV3p, FILp and WUSp) isolated from shoot apical meristems of *A. thaliana*. The study isolated protoplasts of the cells by using fluorescent markers unique to them, and revealed a strong expression of the *LFY* gene across all cell types.

During preparation for NCA, three of the TFs (FD, WUS and BLR) and their corresponding gene connections had to be removed as they were not NCA-compliant. The final NCA-compliant network consisted of the remaining 7 TFs and 55 genes. For the NCA, we assumed same connectivity strengths for TF with their target genes across all cell lines, which is a reasonable assumption. NCA provided CS for all TF-gene pairs. However, after NCA decomposition, the CS needed to be checked for their signs (a positive sign signifies activation and a negative sign signifies repression). This is done by comparing the CS with the initial connectivity matrix, and especially the connectivity directions of well-established TF-gene pairs. We found that the TF activities and CS for the AG, HY5, SEP3 and AP2 TFs needed to be corrected for their signs. The TF-gene pairs showing strong CS represent strong binding between a TF and its target. However, many TF-gene pairs showed very low CS, so that their documented regulatory connection would be worth re-examining [26]. Interestingly, AGRIS did not list the direction of interaction between AGL15 and four of the genes regulated by it (*AGL22, AGL25, EDF4* and *RAV2*). NCA deduced AGL15 to be a strong repressor of *AGL22*, strong activator of *RAV2*, moderate activator for *AGL25* and very weak repressor for *EDF4*. Thus, given verified information about the sign of a TF-gene interaction, NCA can deduce whether the TF is an activator or repressor of other target genes based on gene expression data. We should point out though that the strength of NCA is the deduction of quantitative information about a TF-GRN based on verified information about the underlying connections and gene expression data for the network. *AGL22*, also known as Short Vegetative Phase (*SVP*) encodes a TF that can repress flowering time in addition to other genes *AGL15, AGL18* and *FLM*[48-50]. Based on our NCA, we determined that *AGL22* is repressed much more strongly by AGL15 compared to SEP3. Interestingly, though, the gene expression of *AGL22* increased several-fold compared to the control across all four cell types. This might be explained by the observation that even though the TF activity of SEP3 increases relative to the control, the TF activity of AGL15 is reduced compared to the control by a similar extent. As AGL15 controls the repression of *AGL22* more strongly compared to SEP3, the gene expression of *AGL22* compared to the control increases. Two other genes, *HLH1* and *RD20*, are regulated by the same TFs, HY5 (activation) and AP2 (repression). NCA determined *HLH1* to have similar connectivity strengths to both HY5 and AP2 but of opposite signs while *HLH1* gene expression was found to be slightly higher compared to the control strain. This could be because of the slightly higher TF activity of HY5 compared to AP2 as deduced by NCA. *RD20*, on the other hand, was found to be mildly repressed across the four cell types compared to the control. This could be because it is more strongly repressed by AP2 compared to activation by HY5.

Of the different TFs included in our study, LFY plays the role of master regulator during floral development. Out of the direct targets of LFY included in our network, *MYB17* or late meristem identity 2 is very important in meristem identity transition [51]. *MYB17* was found to be very strongly activated by LFY. This, combined with high TF activity of LFY would explain the high expression levels seen for the *MYB17* gene from mRNA analysis. We were unable to include AP1, which is another important TF in the meristem identity pathway that is known to interact in a positive feedback network with LFY and MYB17. We can, however, deduce that the AP1 TF would have higher activity across the four cell types compared to the control based on strong activities of LFY and MYB17. In fact, the reproductive phase in Arabidopsis involves the transition of the SAM to an inflorescence meristem and then to a floral meristem [44]. The floral meristem identity proteins in Arabidopsis [44] include the TFs that were found to be upregulated from our analysis (LFY and SEP3) which seems to indicate that the cells were isolated from a floral and not a vegetative meristem.

We compared the TF activities obtained by NCA with the expression values for their corresponding genes. TF activities can in general be expected to be proportional to the expression levels of the corresponding genes. However, TFs that need to undergo extensive post-translational modification to be active can be exceptions to this expected trend. Our analysis showed that the profiles of TF activities obtained from NCA compared well with the expression levels of the genes coding for these TFs in the case of the majority of TFs (LFY, AG, HY5, AP3/PI and SEP3 (in two out of four cell types). However AP2 and AGL15 are exceptions. The discrepancy for AP2 and AGL15 could quite possibly be because of the large error in the measurement of the microarray replicates leading to problems with the NCA. A repeat of the gene expression analysis with better control on the replicates may provide a better answer to this. If a discrepancy is still observed, this would indicate a change in TFs due to post-transcriptional and post-translational modifications. NCA thus allows the generation of newer hypotheses relating to the conversion of a gene product to an active TF based on how well the gene expression results agree with the deduced activities of their corresponding TFs. As a further step, we compared normalized values for both, using maximum or minimum values for TF activity or gene expression across the four cell types to allow better comparison between them. We found a very good correlation for LFY; decent matches for AG, SEP3, HY5 and AP3/PI; and poor matches for AP2 and AGL15 from this analysis.

The application of NCA to microbial and mammalian systems has provided interesting insights into gene regulation by TFs. As previously described, the applications of NCA to microbial systems include the following: (i) investigation of TF changes during cell cycle regulation in *S. cerevisiae*[26] (ii) analysis of changes in TF activities in *E. coli* during the change from a glycolytic carbon source (glucose) to a gluconeogenic carbon source (acetate) [27] (iii) studying the effects of reactive nitrogen species on a TF network in *E. coli*[31,32] (iv) identification of TFs important in the isobutanol response network in *E. coli*[33] and (v) determining TF-gene interactions in *B. subtilis* during a carbon source transition from glucose to malate and vice-versa [34], Applications of NCA to mammalian systems are more recent (i) studying the effects of overexpression of the glycerol kinase gene in rat hepatoma cells [35] and (ii) identifying TFs with altered activity in response to PTEN expression [37].

These studies of TF-GRNs have revealed the strengths of NCA in providing insights about the regulatory aspects of a system given the basic structural information about the underlying network. In the case of plants, there is lesser information available about TF-gene interactions. The AtRegNet database from AGRIS, which is the most comprehensive resource for such information, contains 768 confirmed TF-gene interactions for 46 TFs in *A. thaliana*, which is estimated to contain more than 1700 TFs [52]. In our NCA of a network derived from AGRIS, the original network consisting of 10 TFs and 57 genes reduced to 7 TFs and 55 genes for NCA compliance. This is because of the absence of sufficient regulatory information about the three TFs that had to be removed. NCA requires that any TF in a network regulate at least two genes. The availability of more information about TF-gene interactions would overcome this issue of NCA non-compliant TFs.

NCA uses gene expression data and underlying network connectivity during its analysis; consequently, the quantitative measures provided by NCA are dependent on the accuracy of the underlying network. For example, many of the genes considered in this study have unconfirmed interactions with other TFs. If any of these interactions were confirmed, the current NCA could be rerun to account for the effect of additional TFs on expression of the target genes. Thus, having correct prior connectivity information about a network would increase the accuracy of NCA substantially. Such information on TF-gene interactions is obtained mainly through ChIP-CHIP or ChIP-SEQ experiments that allow the detection of binding patterns of TFs with DNA sequences. In fact, a lot of the confirmed interactions between TFs and genes listed on AGRIS are derived from such papers investigating binding targets for particular TFs [19].

Another limitation of NCA is its inability to model feedback and feedforward regulations between TFs. TF-GRNs are cascades of TFs regulating genes where the product of many genes are TFs that regulate downstream genes. However, for NCA, if a TF is included as a regulator in a network, the gene corresponding to it cannot be included in the network. As a result, NCA cannot determine how strongly other TFs influence the expression of the corresponding gene. In our original network, AG was included as a TF and also present as a gene regulated by LFY, AG, SEP3, AP2, WUS and BLR. We had to remove the AG gene during the NCA because of the presence of AG as a regulatory TF. This limits the application of NCA to non TF target genes in many instances.

Additionally, the NCA decomposition suffers from some variability in estimating CS and TF activity from gene expression data. This is because the NCA decomposition is unique to a scaling factor which can be different for each TF and vary during different data decomposition of the same set of gene expression values and initial connectivity matrix. NCA uses a two-step least squares approach to minimize the difference between experimental and NCA reconstructed gene expression data. As a result, based on the scaling factor chosen, the same gene expression data and initial connectivity matrix could give slightly differing TF activities and CS. In addition, the decomposition process might introduce some variability in estimating TF activities and CS. For the NCA decomposition of the floral TF-GRN used in this study, we found differences in TF activities and CS during repeat runs (Additional file 3). For this network, the LFY TF shows very little variability across the different runs while the other TFs have greater degree of variability. Thus, while the TF activity and CS obtained from NCA decomposition provide quantitative measures for the underlying network, they should be treated not as absolute but relative parameters.

Another drawback that all approaches for modeling gene expression of eukaryotic organisms suffer from, is the inability to include all the factors that regulate gene expression [53]. Most of the current modeling approaches depict gene expression to result from the effect of some of these factors alone, which is not the case [5]. For example, microRNAs play a very important role in gene regulation at the post-transcriptional level similar to the TF regulation at the transcriptional level [54-56]. In humans, microRNAs have been found to use two modes for gene regulation – the first mode is rapid and modulated by homoclusters; the second is delayed and mediated by heteroclusters of microRNAs. Of the two, heteroclusters have been found to indirectly influence gene regulation in tandem with TFs [54]. In addition to microRNAs, other factors including chromatin structure and nucleosome sliding would affect gene expression especially in eukaryotes [53]. Consequently, an accurate model for depicting gene regulation in eukaryotes would have to include all these interactions to capture the true picture of genetic regulation.

Despite these limitations, NCA can provide very interesting hypotheses and insights about regulatory signals in a TF-GRN. Previous applications have shown its utility in understanding microbial systems whose regulatory networks are well characterized, and mammalian sytems to some extent. Plants and eukaryotes operate more complex regulatory mechanisms. Additionally, complicated post-translational modifications can alter the activity of a TF compared to its mRNA transcript level. Consequently, the application of NCA to plant systems would provide interesting insights about these. Hence, there is a need for applying significant efforts in obtaining information about interactions between TFs and genes in plants for constructing TF-GRNs. Such information coupled with NCA would allow the determination of underlying properties of the system and establish paradigms for predicting cellular behavior.

## Conclusions

In this work, we applied constructed a plant TF-GRN important in flower development using regulatory information from the AGRIS database. The initial network consisting of 10 TFs and 57 genes was found to be NCA-compliant for 7 TFs and 55 genes. We applied NCA to the reduced network to obtain CS between TF-gene pairs and TF activities. The CS showed strong connectivity between certain TF-gene pairs including LFY → *MYB17*, LFY → *TLP8*, AP2 → *HLH1*, AP2 → *RD20*, AGL15 → *AGL22*, AGL15 → *RAV2*, HY5 → *HLH1* and HY5 → *RD20*, among others. For some of the co-regulated genes, we were able to determine the extent of transcriptional control of different TFs on a target gene using the CS. Additionally, we were able to determine TF activities for all TFs. Good agreement was seen for the changes in TF activities for multiple TFs and their corresponding gene expression levels. However, for some of the TFs (AP2, SEP3 and AGL15), the change in TF activities did not match with changes in gene expression levels. There could be multiple reasons for this discrepancy including post translation modifications which significantly alter the activity of a TF; noisy data or the small size of the network among others.

Our study is the first application of NCA to a plant TF-GRN and demonstrates the power of NCA for determining nontrivial information about a network based solely on gene expression data and underlying network connectivity. NCA has been widely used to decipher interesting insights about microbial TF-GRNs. However, since NCA relies on underlying network connectivity, incomplete information about the network hinders the accuracy of NCA. Plant TF-GRNs are poorly documented with sparse data about specific sets of TFs and processes. As more information about TF-GRNs is uncovered in plants, similar analysis using NCA would provide profound insights regarding the role of TFs in various cellular processes.

## Methods

### TF-gene network reconstruction

We obtained TF-gene connectivity information from AGRIS (http://arabidopsis.med.ohio-state.edu) [19]. For the GRN analysis, we selected 10 TFs known to be important in floral development and listed in AGRIS. We selected 57 genes that were documented in AGRIS to be the targets of these TFs (Additional file 1, Sheet: AGRIS TF-gene verification). We constructed an initial connectivity matrix to map the TF-gene interactions documented in AGRIS (Additional file 1, Sheet: Initial connectivity matrix). Entries in this matrix were 1 (indicating a documented activation interaction), –1 (indicating a documented repression interaction) or 0 (indicating no documented interaction). Documented TF-gene interactions for which the type of interaction (activation or repression) were not known were assigned an entry of 1 (highlighted cells).

### Gene expression data

We used the Botany Array Resource (http://www.bar.utoronto.ca) [39] for obtaining gene expression data pertinent to the TFs and genes in our network during floral development. This database provided gene expression data from the study by Yadav *et al.*[38] that provided expression levels of the genes of interest across four SAM cell types. The original and log transformed gene expression data are summarized in Additional file 1 (Sheet: Original microarray data, and Sheet: Log transformed microarray data, respectively).

### NCA

We used the NCA toolbox (http://www.seas.ucla.edu/~liaoj/downloads.html) [26,30] in conjunction with the initial TF-gene connectivity matrix (Additional file 1, Sheet: Initial connectivity matrix) for decomposing the gene expression data. We independently analyzed the gene expression dataset corresponding to each biological replicate of each cell line. On completion, NCA provided TF activities for each replicate of each cell line (Additional file 2, Sheet: TFA and mRNA) as well as TF-gene CS common to all cell lines (Additional file 2, Sheet: Connectivity strengths).

## Competing interests

The authors declare no financial or non-financial competing interests.

## Authors’ contributions

AM and GS conceived the study. AM collected and analyzed the data, AM and GS compiled and interpreted the results. AM and GS drafted the manuscript. GS revised the manuscript. Both authors read and approved the final manuscript.

## Supplementary Material

Additional file 1 **Input data for NCA.** Gene reference sheet: Gene models for the genes analyzed in this study, their common names and the number used to represent them in Figures 1 and 2. Initial connectivity matrix sheet: Matrix of connectivity information obtained between TFs and target genes from AGRIS. AGRIS TF-gene verification sheet: Data retrieved from AGRIS for constructing initial connectivity matrix. Original microarray data sheet: Microarray data retrieved for all the genes in this study across four different cell types (named CLV3n, CLV3p, FILp and WUSp) derived from shoot apical meristems of *A. thaliana* using the Botany Array Resource. Click here for file

Additional file 2 **Output data from NCA.** NCA-compliant network sheet: TFs and genes compliant for NCA obtained by initial NCA feasibility analysis. Connectivity strengths sheet: CS obtained by NCA. As NCA may invert the sign for the CS during the decomposition, CS for some of the TFs had to be corrected based on well-established TF-gene connectivity information. Gene expression sheet: Log_10_ fold expression changes of genes obtained from microarray data and NCA simulated expression data. TFA and mRNA sheet: Log_10_ fold changes in TF activities compared to control obtained by NCA and corresponding changes in mRNA values for all four cell types included in the study. Activities for some of the TFs had to be corrected in their sign based on the changes for the CS previously mentioned. Normalized TFA and mRNA sheet: Calculation of normalized TF activity and mRNA levels from the average TF activities and mRNA levels across all four cell types (expressed as log_10_ fold changes compared to control). Click here for file

Additional file 3 **Identifiability of NCA results: variability in estimating TF and CS from same gene expression data and initial connectivity strengths.** TF activities and CS obtained in five independent executions of NCA from the same gene expression data and initial connectivity matrix used in this study. Click here for file
